# Leveraging generative AI to assist biocuration of medical actions for rare disease

**DOI:** 10.1093/bioadv/vbaf141

**Published:** 2025-06-12

**Authors:** Enock Niyonkuru, J Harry Caufield, Leigh C Carmody, Michael A Gargano, Sabrina Toro, Patricia L Whetzel, Hannah Blau, Mauricio Soto Gomez, Elena Casiraghi, Leonardo Chimirri, Justin T Reese, Giorgio Valentini, Melissa A Haendel, Christopher J Mungall, Peter N Robinson

**Affiliations:** Trinity College, Hartford, CT 06106, United States; The Jackson Laboratory for Genomic Medicine, Farmington, CT 06032, United States; Division of Environmental Genomics and Systems Biology, Lawrence Berkeley National Laboratory, Berkeley, CA 94720, United States; Division of Environmental Genomics and Systems Biology, Lawrence Berkeley National Laboratory, Berkeley, CA 94720, United States; The Jackson Laboratory for Genomic Medicine, Farmington, CT 06032, United States; The Jackson Laboratory for Genomic Medicine, Farmington, CT 06032, United States; Department of Genetics, The University of North Carolina at Chapel Hill, Chapel Hill, NC 27599, United States; Department of Genetics, The University of North Carolina at Chapel Hill, Chapel Hill, NC 27599, United States; The Jackson Laboratory for Genomic Medicine, Farmington, CT 06032, United States; AnacletoLab, Computer Science Department, Dipartimento di Informatica, Università degli Studi di Milano, Milan 20133, Italy; Division of Environmental Genomics and Systems Biology, Lawrence Berkeley National Laboratory, Berkeley, CA 94720, United States; AnacletoLab, Computer Science Department, Dipartimento di Informatica, Università degli Studi di Milano, Milan 20133, Italy; Berlin Institute of Health at Charité—Universitätsmedizin Berlin, Berlin 10117, Germany; Division of Environmental Genomics and Systems Biology, Lawrence Berkeley National Laboratory, Berkeley, CA 94720, United States; AnacletoLab, Computer Science Department, Dipartimento di Informatica, Università degli Studi di Milano, Milan 20133, Italy; Department of Genetics, The University of North Carolina at Chapel Hill, Chapel Hill, NC 27599, United States; Division of Environmental Genomics and Systems Biology, Lawrence Berkeley National Laboratory, Berkeley, CA 94720, United States; The Jackson Laboratory for Genomic Medicine, Farmington, CT 06032, United States; Berlin Institute of Health at Charité—Universitätsmedizin Berlin, Berlin 10117, Germany

## Abstract

**Motivation:**

Structured representations of clinical data can support computational analysis of individuals and cohorts, and ontologies representing disease entities and phenotypic abnormalities are now commonly used for translational research. The Medical Action Ontology (MAxO) provides a computational representation of treatments and other actions taken for clinical management. Currently, manual biocuration is used to annotate MAxO terms to rare diseases. However, it is challenging to scale manual curation to comprehensively capture information about medical actions for the more than 10 000 rare diseases.

**Results:**

We present AutoMAxO, a semi-automated workflow that leverages Large Language Models (LLMs) to streamline MAxO biocuration. AutoMAxO first uses LLMs to retrieve candidate curations from abstracts of relevant publications. Next, the candidate curations are matched to ontology terms from MAxO, Human Phenotype Ontology (HPO), and MONDO disease ontology via a combination of LLMs and post-processing techniques. Finally, the matched terms are presented in a structured form to a human curator for approval. We used this approach to process abstracts related to 37 rare genetic diseases and identified 958 novel treatment annotations that were transferred to the MAxO annotation dataset.

**Availability and implementation:**

AutoMAxO is a Python package freely available at https://github.com/monarch-initiative/automaxo.

## 1 Introduction

Rare diseases present significant challenges in healthcare due to their low prevalence and high complexity. While individual rare diseases have low prevalence, collectively they affect approximately one in 10 Americans and nearly 300 million people globally (Tisdale *et al.* 2021). Most rare conditions are difficult to diagnose, with many patients waiting years for an accurate diagnosis. Despite over 10 000 rare diseases having been identified, fewer than 5% have FDA-approved treatments (Haendel *et al.* 2020, Fermaglich and Miller 2023). Clinicians and researchers depend on various resources such as GeneReviews, primary literature, clinical trial databases, and other published medical literature to identify potential treatments. These resources, however, present a disjointed landscape that can be burdensome to navigate effectively. Biomedical ontologies are structured frameworks that categorize medical concepts to enable sophisticated searching and algorithmic analysis and can thereby support clinicians and researchers in finding relevant information.

Large language models (LLMs) are advanced AI models trained on extensive text data to interpret and generate human language. These models use deep learning techniques to produce contextually relevant and coherent text, demonstrating versatility in translation, summarization, and question-answering tasks. LLMs have been applied to numerous medical domains (Singhal *et al.* 2023). Biocuration, the process of collecting, organizing, and annotating biological data, ensures the accuracy and usefulness of information for research and clinical applications. In the realm of biological research, accurate data curation is crucial for meaningful scientific advancements. Biocurators manually review literature and databases to extract data about genes, proteins, diseases, and phenotypes, organizing it into structured ontologies (Howe *et al.* 2008). However, biocuration requires significant human effort, highlighting the need for automated solutions to enhance efficiency in biocuration (Singhal *et al.* 2016).

To reduce the workload of manual curators, various efforts have developed methods for completing information extraction tasks with LLMs. Agarwal *et al.* found that OpenAI’s GPT-3 model could accurately complete specific extraction tasks, such as abbreviation expansion and medication attribute extraction, from clinical text with no specific training. They also found that prompts directing the LLM to yield a specific output structure improved performance in resolving results (Agrawal *et al.* 2022). Subsequently, information extraction researchers have assembled both new benchmark sets [e.g. MultiMedQA (Singhal *et al.* 2023), BioLLMBench (Sarwal *et al.* 2023)] and domain-adapted models [e.g. Med-PaLM (Singhal *et al.* 2023), Med-MLLM (Liu *et al.* 2023), and BioMistral (Labrak *et al.* 2024)] to extract an increasingly broad range of data elements from medical text. SPIRES (Structured Prompt Interrogation and Recursive Extraction of Semantics) (J. H. Caufield *et al.* 2024) is a knowledge extraction method that leverages both LLMs and ontologies. SPIRES uses knowledge schemas defined using LinkML (Moxon *et al.* 2021) to extract entities and relationships from text. Since each schema encapsulates specific domain concepts, relationships, and properties, it can be used to craft more effective prompts for LLMs, therefore obtaining reliable annotations in the form of textual elements. A key aspect of SPIRES’ effectiveness is its ability to ground these textual elements as concepts derived from ontologies (i.e. to identify ontology terms that match the concepts identified by the LLM), including Open Biological and Biomedical Ontologies (OBO) Foundry ontologies (Jackson *et al.* 2021).

The Medical Action Ontology (MAxO) provides a computational representation of medical diagnostics, preventions, procedures, interventions, and therapies. MAxO follows OBO standards, with terms having unique identifiers, names/labels definitions, in addition to computational logical definitions, and synonyms that can be used for NLP applications. A medical action is broadly regarded as any medical procedure, intervention, therapy, and or measurement undertaken for clinical management. The structure of MAxO is composed of six upper level terms, viz., diagnostic procedure, preventative therapy, therapeutic procedure, medical action avoidance, palliative care, and complementary and alternative medical therapy (Carmody *et al.* 2023). Currently, MAxO includes 1902 medical action terms, curated through manual and semi-automated methods. MAxO is compatible with other ontologies within the OBO Foundry (Jackson *et al.* 2021), enhancing the ability to model diseases and phenotypic features comprehensively. It provides a computational representation of treatments and actions for clinical patient management and is integrated with the Mondo Disease Ontology (Mondo) and the Human Phenotype Ontology (HPO), broadening the scope of computational disease modeling of rare diseases (Carmody *et al.* 2023).

Like many ontologies, in OBO, MAxO is used for the annotation of knowledge curated from the literature. The MAxO annotation model is designed to systematically describe medical actions and their relationships to diseases and phenotypic features. It enables a structured and interoperable way to represent clinical interventions and management strategies within biomedical research and healthcare by capturing relationships between disease, phenotypes, and medical action. The core MAxO annotation model relates medical actions to phenotypes in the context of a disease For example:

Medical Action: *copper chelator agent therapy [MAXO : 0001224]*

Relationship: PREVENTS

Phenotype: Cirrhosis [HP : 0001394]

Disease: *Wilson disease Anemia [MONDO : 0010200]*

Identifiers are used for all concepts to avoid ambiguity. MAxO annotations are disease-specific. In this example, copper chelator agent therapy is indicated to prevent Cirrhosis in the context of Wilson disease Anemia. However, *copper chelator agent therapy [MAXO : 0001224]* would not be indicated in other diseases characterized by *Cirrhosis [HP : 0001394]*, such as *Hepatitis C [MONDO : 0005231], Primary Biliary Cholangitis [MONDO : 0009588]* or *Alcoholic Liver Disease [MONDO : 0043693]*

The MAxO annotation model is currently being mapped to the Biolink Model, to allow for inclusion into Knowledge Graphs (KG), such as the NCATS Translator KG, Monarch KG, and KG-Hub, and to allow graph machine learning methods such as link prediction to apply to these data using software such as GRAPE.

**Table 3.** Summary of the counts of novel annotations derived with automaxo.^a^

MONDO	Annotations	MAxO (*n*)	HPO (*n*)	Example MAxO	Example relation	Example HPO
Achondroplasia (MONDO : 0007037)	2	1	1	human growth hormone replacement therapy (MAXO : 0000780)	prevents	Short stature (HP : 0004322)
alkaptonuria (MONDO : 0008753)	13	7	6	pharmacotherapy (MAXO : 0000058)	treats	Elevated urinary homogentisic acid (HP : 0033704)
Apert syndrome (MONDO : 0007041)	24	11	15	airway management (MAXO : 0000500)	treats	Apert syndrome (MONDO : 0007041)
Ataxia telangiectasia (MONDO : 0008840)	10	8	5	corticosteroid agent therapy (MAXO : 0000640)	treats	Phenotypic abnormality (HP : 0000118)
Brugada syndrome (MONDO : 0015263)	10	3	6	ablation therapy (MAXO : 0000452)	prevents	Ventricular arrhythmia (HP : 0004308)
Camurati-Engelmann disease (MONDO : 0007542)	10	7	5	corticosteroid agent therapy (MAXO : 0000640)	treats	Bone pain (HP : 0002653)
Canavan disease (MONDO : 0010079)	8	4	4	gene therapy (MAXO : 0001001)	treats	Canavan disease (MONDO : 0010079)
celiac disease (MONDO : 0005130)	67	1	8	dietary gluten intake avoidance (MAXO : 0010000)	prevents	celiac disease (MONDO : 0005130)
Chediak-Higashi syndrome (MONDO : 0008963)	17	3	2	allogeneic hematopoietic stem cell transplantation (MAXO : 0001479)	treats	Chediak-Higashi syndrome (MONDO : 0008963)
citrullinemia, type II, adult-onset (MONDO : 0011326)	14	5	3	carbohydrate-restricted diet intake (MAXO : 0000771)	treats	Hepatic encephalopathy (HP : 0002480)
Donnai-Barrow syndrome (MONDO : 0009104)	8	7	8	surgical procedure (MAXO : 0000004)	treats	Congenital diaphragmatic hernia (HP : 0000776)
Dravet syndrome (MONDO : 0100135)	46	5	4	anticonvulsant agent therapy (MAXO : 0000167)	prevents	Seizure (HP : 0001250)
Familial Mediterranean fever (MONDO : 0018088)	36	9	5	interleukin-1 receptor inhibitor therapy(MAXO : 0001488)	treats	Phenotypic abnormality (HP : 0000118)
Fanconi anemia (MONDO : 0019391)	53	10	8	hematopoietic stem cell transplantation (MAXO : 0000747)	treats	Bone marrow hypocellularity (HP : 0005528)
Gaucher disease (MONDO : 0018150)	48	8	6	enzyme replacement or supplementation therapy (MAXO : 0000933)	prevents	Phenotypic abnormality (HP : 0000118)
Gaucher disease type II (MONDO : 0009266)	3	1	1	enzyme replacement or supplementation therapy (MAXO : 0000933)	treats	Phenotypic abnormality (HP : 0000118)
Gaucher disease type III (MONDO : 0009267)	6	3	3	diuretic agent therapy (MAXO : 0000165)	treats	Restrictive cardiomyopathy (HP : 0001723)
Huntington disease (MONDO : 0007739)	46	22	16	palliative care (MAXO : 0000021)	treats	Huntington disease (MONDO : 0007739)
hypochondroplasia (MONDO : 0007793)	4	3	3	human growth hormone replacement therapy (MAXO : 0000780)	treats	Short stature (HP : 0004322)
Lesch-Nyhan syndrome (MONDO : 0010298)	21	9	9	umbilical cord blood transplantation (MAXO : 0010033)	treats	Lesch-Nyhan syndrome (MONDO : 0010298)
Loeys-Dietz syndrome (MONDO : 0018954)	20	11	15	gastrostomy (MAXO : 0001346)	treats	Failure to thrive (HP : 0001508)
lymphatic malformation 1 (MONDO : 0007919)	3	3	3	physical therapy (MAXO : 0000011)	treats	Lymphedema (HP : 0001004)
Maple syrup urine disease (MONDO : 0009563)	53	10	3	dietary branched-chain amino acid intake avoidance (MAXO : 0010102)	prevents	Phenotypic abnormality (HP : 0000118)
Marfan syndrome (MONDO : 0007947)	44	17	18	angiotensin receptor blocker therapy (MAXO : 0000653)	prevents	Aortic root aneurysm (HP : 0002616)
Mucopolysaccharidosis type 1 (MONDO : 0001586)	48	7	13	corneal transplantation (MAXO : 0010034)	treats	Corneal opacity (HP : 0007957)
Noonan syndrome (MONDO : 0018997)	31	17	22	behavioral dietary intervention (MAXO : 0000884)	treats	Gastroparesis (HP : 0002578)
Primary ciliary dyskinesia (MONDO : 0016575)	24	13	8	proton pump inhibitor agent therapy (MAXO : 0000272)	treats	Peptic ulcer (HP : 0004398)
propionic acidemia (MONDO : 0011628)	22	8	4	pharmacotherapy (MAXO : 0000058)	treats	Acute hyperammonemia (HP : 0008281)
Rett syndrome (MONDO : 0010726)	12	9	3	deep brain stimulation (MAXO : 0000943)	treats	Phenotypic abnormality (HP : 0000118)
sickle cell anemia (MONDO : 0011382)	117	31	21	gene therapy (MAXO : 0001001)	treats	sickle cell anemia (MONDO : 0011382)
Spinal muscular atrophy(MONDO : 0001516)	39	6	4	antisense oligonucleotide inhibitor therapy (MAXO : 0001593)	treats	Phenotypic abnormality (HP : 0000118)
Spinal muscular atrophy, type 1 (MONDO : 0009669)	2	2	1	gene therapy (MAXO : 0001001)	treats	Phenotypic abnormality (HP : 0000118)
Spinal muscular atrophy, type II (MONDO : 0009673)	1	1	1	biologic therapy (MAXO : 0000015)	treats	Decreased muscle mass (HP : 0003199)
Spinal muscular atrophy, type III (MONDO : 0009672)	2	2	2	vaccination (MAXO : 0001017)	prevents	Phenotypic abnormality (HP : 0000118)
Stickler syndrome (MONDO : 0019354)	11	6	5	vitrectomy (MAXO : 0001085)	treats	Rhegmatogenous retinal detachment (HP : 0012230)
Tuberous sclerosis (MONDO : 0001734)	35	15	12	laser ablation therapy (MAXO : 0000453)	treats	Adenoma sebaceum (HP : 0009720)
Wilson disease (MONDO : 0010200)	48	9	16	liver transplantation (MAXO : 0001175)	treats	Hepatic failure (HP : 0001399)

a 958 novel annotations were obtained for a total of 37 diseases that involved a total of 269 unique HPO terms and 294 unique MAxO terms. One example annotation is shown for each disease.

**Figure 1.**
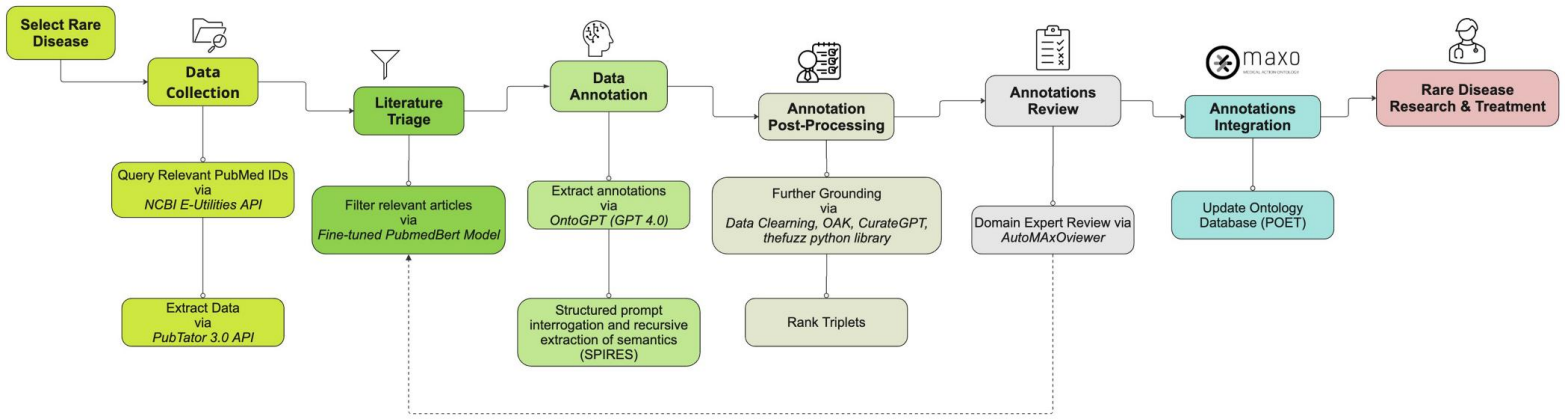
Workflow Diagram for AutoMAxO Project. This diagram illustrates the step-by-step process of the AutoMAxO project, from selecting rare diseases and collecting data to processing, validating, and integrating data into the MAxO database for enhancing rare disease treatment.

Currently, MAxO annotation is a manual process, involving an expert curator selecting and carefully reading relevant papers and manually entering annotations via a specialized tool. We present AutoMAxO, a semi-automated workflow that leverages LLMs and SPIRES to assist with the annotation and update of the MAxO ontology for rare diseases. The summary of the workflow is shown in Fig. 1.

## 2 Methods

### 2.1 Automated retrieval of relevant text (data collection)

To automatically mine the relevant literature from PubMed, AutoMAxO either uses a list of PubMed identifiers (PMID) representing published articles selected by the users or exploits a selection of MeSH codes representing diseases of interest and then employs the NCBI E-Utilities API (Sayers *et al.* 2022) to gather PubMed IDs of peer-reviewed articles focused on these diseases. By using specific search criteria, the user may tailor the search to, for example, retrieve articles related to potential therapies and then filter them to obtain a maximum number of papers that is below a (user-set) value. The paper filtering process is chosen by the user, who may select only publications related to, e.g. disease keywords and MeSH tree mappings of interest, or to prioritize the retrieval of publications that are within time ranges or being the most relevant (highly cited). Importantly, all the PubMed IDs of the papers extracted based on a user query are saved so that successive, potentially different, queries will retrieve them; this guarantees that each run of AutoMAxO processes a set of “unseen” papers.

After retrieving relevant PubMed IDs from E-Utilities, AutoMAxO uses PubTator 3.0 API to retrieve titles and abstracts corresponding to the PubMed IDs (Wei *et al.* 2024). AutoMAxO saves the texts in a comma-separated values (CSV) file, to be used as input for the next step of LLM-driven parsing.

### 2.2 Literature triage

We observed that despite having filters to select only articles related to treatments for specific diseases, we still retrieved some articles outside the scope of MAxO, such as those focused on model organisms like mice. To reduce the number of out-of-scope articles, we therefore fine-tuned the PubMedBERT model (Lee *et al.* 2020) to classify articles as either relevant or not relevant.

Initially, all articles related to disease treatments retrieved from E-Utilities were passed through SPIRES and the final pipeline for all 37 diseases. Based on curators’ feedback—accepting or rejecting different annotations as described in the following steps—we further refined the model. Among all the selected papers, we considered as relevant (positive) those that allowed to extract triples expressing relationships between ontology terms; to extract a negative set, we considered those papers that did not produce any meaningful triple and that were also validated as out-of-scope by expert curators. By applying this selection procedure, we identified 493 relevant papers and 225 negative papers.

The fine-tuned model was trained on 80% of the data and tested on 20%, achieving a validation accuracy of 90.4% (F1 score 90.3%, precision 90.3%, recall 90.4%). This model is now used to filter all incoming articles from EUtils before they are passed to SPIRES for ontology extraction. Articles that are classified as relevant are then processed further by OntoGPT.

### 2.3 Data annotation by SPIRES and OntoGPT

For extracting structured information from the text, we utilized OntoGPT Version 0.3.9, a Python package we developed for parsing text with large language models (LLMs), using instruction prompts and ontology-based grounding. Within OntoGPT, we used GPT-4 model version “gpt-4–0125-preview” to extract annotations related to medical actions, relationships with diseases, and phenotypes. OntoGPT implements SPIRES (J. H. Caufield *et al.* 2024), which incorporates a schema and ontology-driven extraction of annotations from text. In this process, using our MAxO schema (https://github.com/monarch-initiative/automaxo/blob/main/src/automaxo/maxo_template.yaml), OntoGPT generates candidate annotations consisting of five elements: subjects (medical actions), predicates (relationships), objects (phenotypes), qualifiers (diseases), and subject extensions (chemical entities). The title and abstract of each article were used as separate inputs for OntoGPT and the extracted data were saved in YAML format. This YAML contains objects conforming to the AutoMAxO schema, using term identifiers from relevant ontologies. Where OntoGPT cannot ground terms in ontologies, it makes placeholder terms (i.e. it reports words or phrases that might correspond to a term from the corresponding target ontology; the curator can replace the placeholder with the term, if one exists, or create a new term request for a concept that is not currently represented in the ontology).

**Figure 2.**
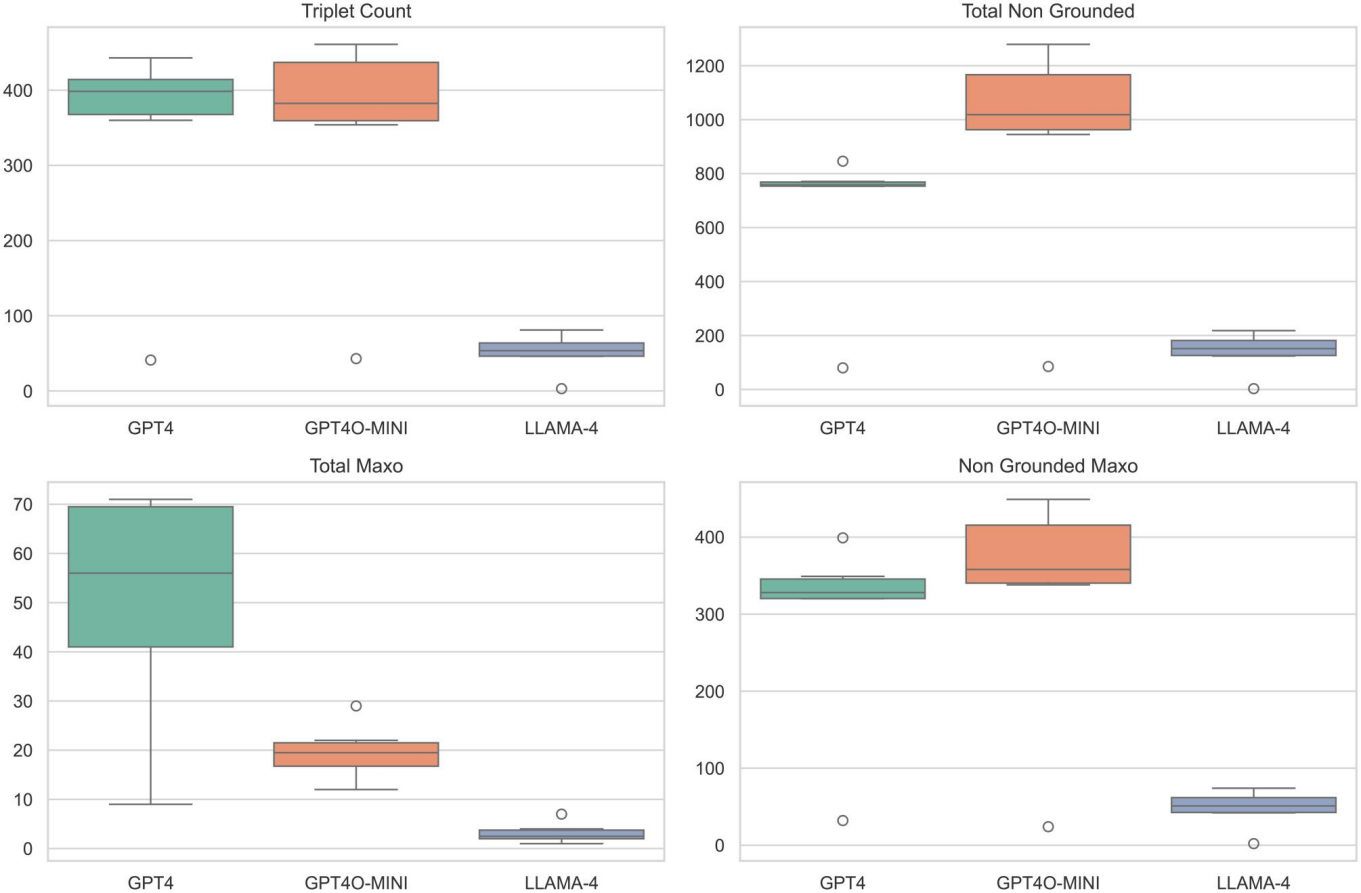
Distribution of LLM performance metrics across six diseases. Each boxplot shows the spread of values for a given metric across GPT-4, GPT-4o Mini, and LLaMA 4. Metrics include the number of extracted triplets, total non-grounded concepts, total MAXO terms, and non-grounded MAXO terms. The central line of each box represents the median; boxes show the interquartile range (IQR), and whiskers extend to 1.5× IQR. These plots highlight consistent underperformance by LLaMA 4, and similar distributions between GPT-4 and GPT-4o Mini.

To justify the use of GPT-4 as our primary model, we conducted a comparative evaluation of three LLMs: GPT-4 (gpt-4–0125-preview), GPT-4o Mini (gpt-4o-mini-2024–07-18), and LLaMA 4. We assessed their performance on six randomly selected diseases using four metrics: the number of extracted triplets, the total number of non-grounded terms, the total number of MAxO terms extracted, and the number of non-grounded MAxO terms, as detailed in Table 1 and Fig. 2. Pairwise Mann–Whitney *U* tests were used to evaluate statistical differences between models across these metrics. As shown in Table 1 and Fig. 2, both GPT-4 and GPT-4o Mini significantly outperformed LLaMA 4 across all metrics (*P* < .05). No statistically significant difference was observed between GPT-4 and GPT-4o Mini for triplet count (*P* = 1.0000) and non-grounded MAxO terms (*P* = .3095), suggesting comparable performance between the two. However, GPT-4 showed a non-significant advantage in minimizing non-grounded outputs overall, which informed our decision to use GPT-4 as the primary model for all downstream annotation tasks.

**Table 1.** Per-disease performance metrics of three large language models (GPT-4, GPT-4o Mini, and LLaMA 4) across six rare diseases.^a^

Disease	Triplet Count			Total Non-Grounded			Total MAxO			Non-Grounded MAxO		
	GPT4	GPT4o-Mini	LLaMA 4	GPT4	GPT4o-Mini	LLaMA 4	GPT4	GPT4o-Mini	LLaMA 4	GPT4	GPT4o-Mini	LLaMA 4
Dravet Syndrome	443	461	65	771	1279	185	44	12	2	399	449	63
Fanconi Anemia	406	389	46	846	1016	124	71	20	4	335	369	42
Hypochondroplasia	41	43	3	80	85	3	9	19	1	32	24	2
Maple Syrup Urine Disease	391	376	47	756	1021	132	70	29	3	321	347	44
Mucopolysaccharidosis Type I	417	453	60	761	1215	171	68	22	2	349	431	58
Rett Syndrome	360	354	81	753	945	218	40	16	7	320	338	74

a For each model, we report the number of extracted triplets, the total number of non-grounded terms, the total number of MAxO terms extracted, and the number of non-grounded MAxO terms. GPT-4 and GPT-4o Mini consistently extract more triplets and MAxO terms and produce fewer non-grounded terms compared to LLaMA 4, reflecting higher grounding reliability and concept coverage.

### 2.4 Annotation post-processing

After OntoGPT processing, each file undergoes further post-processing to ground additional terms that were not initially matched to exact ontology classes in the database. Each term is analyzed to identify close lexical and vector matches between extracted terms and those in MAxO.

During post-processing, we use the annotation capabilities of the Ontology-Access Kit (OAK) to match terms that were not already grounded to existing ontologies, employing lexical matching of characters and words. If an exact match is not found, we use the CurateGPT tool’s ontology embedding functionality to perform vector matching with ontologies in MAxO, HPO, and MONDO (H. Caufield *et al.* 2024).

For instance, OntoGPT may identify *allogeneic bone marrow transplantation* as a potential MAxO term, though it may not have an exact match within MAxO. The combined use of OAK and CurateGPT can help locate a related MAxO term, such as *MAxO : 0010030 (bone marrow transplantation)*. While this is a broader concept than the one extracted, it is still valid for use. After grounding and identifying potential ontology terms, AutoMAxO aggregates and groups all extracted annotations, ranking them based on their frequency of occurrence (confidence score).

**Figure 3.**
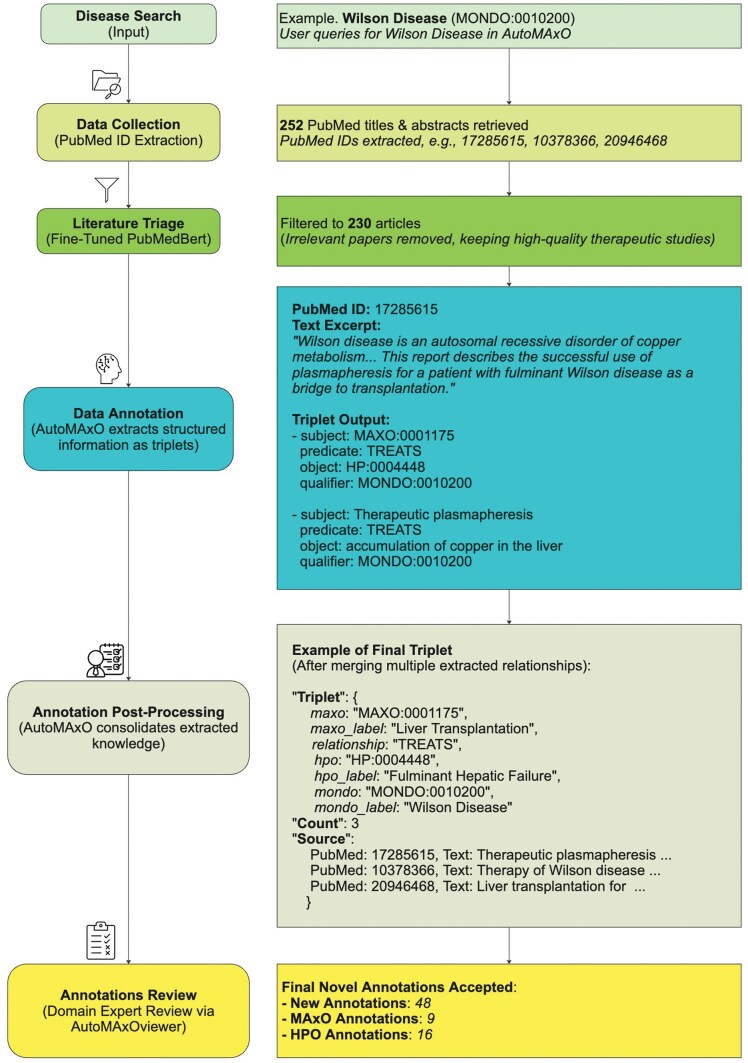
AutoMAxO Workflow: Wilson Disease Case Study. This diagram illustrates the AutoMAxO pipeline using Wilson Disease (MONDO : 0010200) as an example. The workflow follows the process from PubMed article retrieval, filtration, and OntoGPT extraction to structured triple generation, post-processing, and expert review, leading to novel MAxO and HPO annotations for enhanced medical knowledge representation.

For each annotation, AutoMAxO records literature evidence, including PubMed IDs and relevant text excerpts from which the annotations were extracted. All results are saved in a JSON file that can be used by curation tools. By default, AutoMAxO stores results in a folder called “data”, and 20 such folders are included in the GitHub repository as examples. A subfolder is created for each analyzed disease. For instance, one of the subfolders is called alkaptonuria. Each disease folder contains several files created by AutoMAxO. An example of how this step was shown in Fig. 3 for Wilson Disease on the step of Data Post-Processing.

### 2.5 Annotation review using automaxoviewer

**Figure 4.**
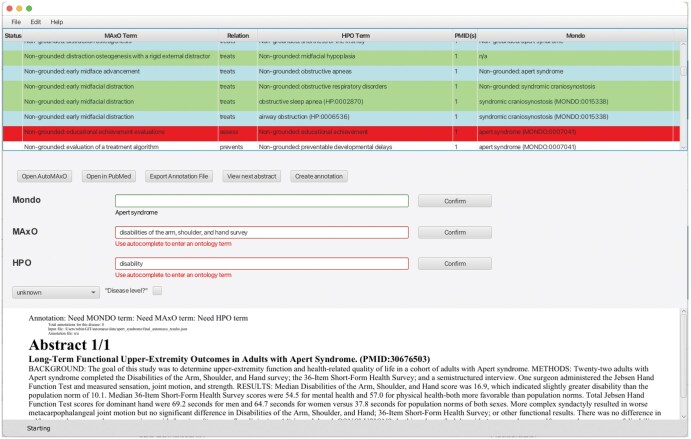
Screenshot of the Automaxoviewer application used to screen the results from AutoMAxO. The last author (PNR; board-certified pediatrician with Habilitation in human genetics) reviewed candidate annotations for relevance and medical correctness.

We created a JavaFX graphical user interface (GUI)-based tool called Automaxoviewer that presents the results of AutoMAxO (from the final_automaxo_results.json file) in tabular form and provides autocomplete and various other functions for a curator to validate and if needed correct or extend the results of AutoMAxO. As shown in Fig. 4, the application allows for streamlined screening and editing of annotations.

The annotations were reviewed by a team of expert curators, including Leigh C. Carmody, Sabrina Toro, and Patricia L. Whetzel. Final validation for all curated annotations was performed by the last author, Peter N. Robinson (PNR), a board-certified pediatrician with a Habilitation in human genetics and over 20 years of experience in medical genetics and biomedical informatics. Due to resource constraints, we were unable to recruit a second physician for independent review; this is a limitation of our current validation process.

For instance, if AutoMAxO is not able to ground a term and provide an exact ontology term, but instead returns a lexical variant (e.g. “liver transpl.” instead of *liver transplantation [MAxO : 0001175])*, the curator can use the autocomplete functionality to assign the MAxO term. The GUI tool simplifies the process of viewing and validating the AutoMAxO results. The Automaxoviewer code is freely available on GitHub under a GNU General Public License version 3 open-source license at https://github.com/monarch-initiative/automaxoviewer.

### 2.6 Annotation integration

The GUI tool outputs a curated annotation file that is ready to be integrated into the “official” MAxO annotations repository using a dedicated script. These annotations form the core of the MAxO ontology database and are accessible at https://poet.jax.org. This curation pipeline ensures that only expert-reviewed annotations are added to the public ontology resource.

## 3 Results

AutoMAxO is designed to streamline the curation of treatments and medical actions for rare diseases by automating ontology-based annotations from biomedical literature. The tool retrieves candidate abstracts from PubMed and leverages OntoGPT to extract structured information using GPT-4. Extracted concepts are mapped to ontology identifiers (grounding) and classified into three categories: True Positives (valid MAxO mappings), False Positives (invalid or non-mappable), and Ambiguous Triples (requiring expert review). This classification refines the extracted annotations, reducing manual workload and extending the functionality of SPIRES. The final candidate annotations are presented to domain experts for validation.

AutoMAxO was evaluated on 37 randomly selected rare genetic diseases, yielding 6299 abstracts, which were processed to extract 15 676 candidate annotations. These annotations were classified into three categories: grounded annotations (successfully mapped to existing ontology terms), possible matches (predicted mappings based on lexical similarity), and unmatched annotations (extracted terms not linked to existing ontologies). Most of the unmatched terms represented false-positive results, but some may represent concepts that could be added to the ontologies. The highest grounding rate was obtained for Mondo concepts (65%), presumably because the abstracts had been chosen based on the corresponding disease. Roughly a quarter of the candidate phenotype annotations were successfully grounded to HPO terms (24%), and 12% of candidate treatment annotations were mapped to MAxO terms.

To assess the quality of the extracted annotations, domain experts reviewed and validated the results using the AutoMAxO Viewer. Annotations were confirmed based on their relevance to case reports, cohort studies, clinical studies, or reviews describing medical actions applied to individuals with the specific disease. Annotations were excluded if they pertained to non-human subjects (e.g. model organisms like mice), irrelevant therapeutic contexts, or were already represented in existing ontologies as synonyms. Additionally, entries were rejected if they contained incorrect relationships, misidentified terms, or focused on genetic or molecular aspects unrelated to clinical management. A summary of this comparison is shown in Table 2.

**Table 2.** Comparison of AutoMaxo-extracted concepts and expert-curated annotations across MAxO, HPO, and total triplet outputs.^a^

Category	AutoMaxo Extracted	Expert Curated	Acceptance Rate
MAXO	1757	294	16.7%
HPO	3570	269	7.5%
Total Triplets	15 676	958	6.1%

a The system extracted over 15 000 triplets, 6.1% of which were ultimately curated and accepted by domain experts.

Following expert review, 958 novel annotations were integrated into MAxO, enhancing its comprehensiveness. The validated annotations have been added to the MAxO annotation GitHub repository for public access. The LLM-assisted approach significantly accelerated ontology curation, allowing curators to process large-scale biomedical literature efficiently while improving ontology coverage and accuracy. A summary of the new annotations is provided in Table 3. The annotations have additionally been added to the MAxO annotation GitHub repository for download.

## 4 Discussion

In this work, we have presented an LLM-based approach to assist biocuration by generating candidate annotations using three current bio-ontologies: MAxO, HPO, and MONDO. AutoMAxO automates several curation tasks that would otherwise be time-consuming and labor-intensive for manual curators, such as identifying abstracts to curate and suggesting relevant ontology terms. We have currently implemented AutoMAxO to emphasize recall over precision, prioritizing the retrieval of a broad set of potentially relevant abstracts. Many of the returned abstracts were related to the disease but did not contain reports of medical actions. For example, some abstracts described research involving model organisms, genetic diagnostics, or other unrelated topics. AutoMAxO Viewer provides a structured interface for curators to efficiently review and filter these abstracts, but full expert review remains necessary to validate the extracted information. It is difficult to objectify the degree of acceleration of curation, but our subjective experience is that the AutoMAxO pipeline substantially improves curation efficiency by quickly pulling in relevant information; when grounding was successful for an abstract, it is relatively easy to assess accuracy and only a click is required to create an annotation. If grounding was not successful, domain knowledge is required to know if the results for an abstract are false-positive or otherwise erroneous and should be skipped or whether the curator should manually search for the required (HPO and MAxO) ontology terms to finalize curation.

The June 2023 release of the MAxO annotation database included 1757 MAxO terms, 18 490 HPO terms, and 411 MONDO terms. With our approach, we curated 958 new annotations that were integrated into the MAxO dataset. While AutoMAxO provides a structured and scalable workflow for candidate annotation generation, we did not perform a direct measurement of time savings compared to manual curation.

Although previous manual curation efforts resulted in 438 annotations over a 5-year period, it is difficult to directly compare annotation rates due to differences in project scope, available resources, and curation methodologies. With AutoMAxO, the candidate annotations for each disease were generated and reviewed in approximately one to two hours per disease. However, we acknowledge that without systematically recorded time data for prior manual efforts, we cannot make direct claims about relative efficiency improvements. AutoMAxO integrates LLMs and advanced search technologies to automate the annotation and updating of medical ontologies. Existing models such as BioBERT(Lee *et al.* 2020) support NLP and curation methods for specific tasks like Named Entity Recognition (NER) and relationship extraction, but they generally need extensive training data to extract medical actions. We are not aware of any other tools currently available tool that specifically retrieves concepts from multiple ontologies (here, MAxO, Mondo, and HPO) that are related by multiple rules (in MAxO, treatments are related to phenotypic features and diseases by the relations treats, prevents, investigates, contraindicated, and lack of observed response).

Additionally, with minimal configuration, AutoMAxO can be adapted to extract annotations from custom text sources, including full-text articles from PubMed Central, websites, or other biomedical text corpora. Its integration with up-to-date ontologies allows for the automatic extraction of structured annotations from the latest research literature, thereby supporting timely and targeted improvements in patient care. While the current implementation focuses on MAxO, the same approach can be applied to other biomedical curation tasks by modifying the SPIRES template file, which defines the extraction schema using ontology terms and relationships.

For researchers interested in adapting this workflow to a different use case, we recommend initially running the pipeline without the triage step. In our case, we included a triage step by fine-tuning PubMedBERT to filter out irrelevant literature, particularly articles focused on mouse models, as our goal was to curate human-specific clinical actions. A similar triage model could be developed using task-specific training data, or simpler rule-based approaches could be implemented—for example, filtering by the presence of keywords related to the target domain.

## 5 Conclusion

In this study, we describe and evaluate AutoMAxO, a workflow for annotating published reports of treatments and other medical actions for rare diseases. AutoMAxO streamlines several time-consuming steps in the process of curation and has enabled us to double the number of annotations for disease-specific MAxO annotations.

## Data Availability

The source code for AutoMAxO is available on GitHub at: https://github.com/monarch-initiative/automaxo. Documentation for AutoMAxO is available here: https://monarch-initiative.github.io/automaxo/. The source code for the AutoMAxO Viewer is available on GitHub at: https://github.com/monarch-initiative/automaxoviewer
